# A SAR Image Target Recognition Approach via Novel SSF-Net Models

**DOI:** 10.1155/2020/8859172

**Published:** 2020-07-09

**Authors:** Wei Wang, Chengwen Zhang, Jinge Tian, Jianping Ou, Ji Li

**Affiliations:** ^1^School of Computer and Communication Engineering, Changsha University of Science and Technology, Changsha 410114, China; ^2^ATR Key Lab., National University of Defense Technology, Changsha 410073, China

## Abstract

With the wide application of high-resolution radar, the application of Radar Automatic Target Recognition (RATR) is increasingly focused on how to quickly and accurately distinguish high-resolution radar targets. Therefore, Synthetic Aperture Radar (SAR) image recognition technology has become one of the research hotspots in this field. Based on the characteristics of SAR images, a Sparse Data Feature Extraction module (SDFE) has been designed, and a new convolutional neural network SSF-Net has been further proposed based on the SDFE module. Meanwhile, in order to improve processing efficiency, the network adopts three methods to classify targets: three Fully Connected (FC) layers, one Fully Connected (FC) layer, and Global Average Pooling (GAP). Among them, the latter two methods have less parameters and computational cost, and they have better real-time performance. The methods were tested on public datasets SAR-SOC and SAR-EOC-1. The experimental results show that the SSF-Net has relatively better robustness and achieves the highest recognition accuracy of 99.55% and 99.50% on SAR-SOC and SAR-EOC-1, respectively, which is 1% higher than the comparison methods on SAR-EOC-1.

## 1. Introduction

Radar Automatic Target Recognition (RATR) technology can achieve the target's attributes, categories, models, and other key characteristics. It can work around the clock and is robust to the environment changes. In order to obtain richer target information from radar signals, RATR technology is increasingly focused on the research of high-resolution radar. Synthetic Aperture Radar (SAR) image is a kind of high-resolution radar image. Compared with High Range Resolution Profile (HRRP), it can provide two-dimensional resolution information of targets and contain more detailed features. However, SAR images are sensitive to the changes of target attitude and speckle noise, which makes it difficult to recognize the SAR targets accurately. So, how to accurately judge the target category of SAR images has become the research focus of RATR technology.

There are two main difficulties existing in SAR image recognition: First, the scattering characteristics between different targets within the same angle may be very similar, which makes it difficult to cluster radar targets. The second is that the geometric structure information hidden in radar images, such as target size and scatter distribution, are complex and nonlinear, which leads to difficulty in information extraction.

Traditional RATR methods include K-nearest neighbor classifier (KNN) and support vector machine learning (SVM). The Principal Component Analysis (PCA) adopted by He et al. [1] has realized the rapid recognition of SAR image targets. Zhao et al. [2] applied SVM to automatic target recognition of SAR images. Trace-norm Regularized multitask learning (Trace), proposed by Obozinskiet et al. [3], assumed that all models share a common low-dimensional subspace, but its method cannot be extended to the nonlinear domain. Evgeniou and Pontil et al. [4] proposed regularized multitask learning (RMTL), which extended the existing kernel based on learning methods for single-task learning, such as SVM. Clustered Multitask Learning (CMTL) approach proposed by Zhou et al. [5] was used to replace Multitask Learning (MTL), which assumed that multiple tasks followed a clustered structure, and it achieved a high accuracy of SAR image recognition. Zhang et al. [6] proposed the Multitask relationship learning (MTRL) approach, which can autonomously learn the positive and negative task correlation, and its recognition accuracy was higher than that of CMTL. Cong et al. [7] proposed a new classification method for clustered multitask learning theory. The method improved MTRL and learned multitask relationships autonomously. It can cluster information of different tasks and easily extended to nonlinear domain.

However, traditional SAR image target recognition technologies often require artificially designing complex feature extraction algorithms, which is difficult to implement and has poor generalization ability. The performance of target recognition algorithm is unstable when the generating environment of radar signal is different. With the development of artificial intelligence, there are more and more applications of target recognition based on deep learning [8]. In the field of optical image recognition, Convolutional Neural Networks (CNNs) have achieved great success. They are widely used in object detection and localization, semantic segmentation, speech recognition, natural language processing, image classification, and target recognition. Compared with other classification algorithms, convolutional neural networks have better robustness for translational changes [9]. Wang et al. [10] proposed a method for SAR image target recognition that combines two-dimensional principal component analysis (2DPCA) and L2 regularization constraint stochastic configuration network (SCN). They applied the 2DPCA method to extract the features of SAR images. Combining 2DPCA and SCN (random learning model with a single hidden layer), the 2DPCA-SCN algorithm have achieved good performance. Due to the limited original SAR images, it is difficult to effectively train the neural networks. In order to solve this problem, multiview deep neural network is proposed by Pei et al. [11]. The framework of this deep neural network includes a parallel network topology with multiple inputs, which can learn the features of SAR images with different views layer by layer. Chen [12] used All Convolutional Neural Network (A-CNN) [13] to the target recognition of SAR images and achieved very high recognition accuracy on the SAR image dataset under standard operating condition, but the recognition performance on SAR image dataset under extended operating condition has declined. Zou et al. [14] proposed another convolutional neural network structure for SAR image target recognition, which uses multiazimuth SAR images to improve the recognition accuracy.

Both the sparsity of SAR images and the limited SAR datasets increase the difficulty of recognition tasks. In response to the above problems, a Sparse Data Feature Extraction (SDFE) module is first designed in this paper. Based on the SDFE module, a small sample sparse data feature extraction network (SSF-Net) is proposed. In order to minimize the network parameters and improve the recognition efficiency, the network has further made improvements of the classifier. The approach in this paper is compared with those in [3–7, 10–12] and achieves higher recognition accuracy and stronger generalization ability.

## 2. SSF-Net Based on SDFE Module

### 2.1. CNNs

**Figure 1:**
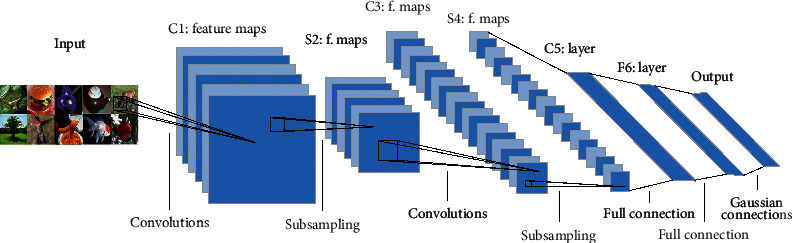
The basic structure of convolution neural network [15].

In recent years, CNN has been widely used in computer vision recognition tasks, and the basis structure of CNN is shown in Figure 1. In 2012, Hinton and Alex Krizhevsky proposed AlexNet [16], which successfully applied ReLU [17], Dropout [18], and LRN [17] in CNN for the first time. Visual geometry group networks (VGGNets) proposed by Simonyan and Zisserman [19] have significantly improved image recognition performance by deepening the network to 19 layers. The application of 3 × 3 small convolution filters is the main contribution of VGGNets. By stacking small convolutional filters, VGGNets not only increases the depth of the network but also enhances the nonlinearity of the convolution layers. Compared with large convolution filters, small filters can also effectively reduce the amount of parameters [20]. Before the VGG network was proposed, An et al. [21] also used small convolution filters, but the network was not as deep as VGGNets. In extracting target features, VGG network has very excellent performance.

Deepening the network will lead to the degradation problems. That is, after sufficient number of training, the accuracy of the training set is saturated or even decreased. In addition, the problems of gradient and information loss also hinder the increase of network depth. Residual net (ResNet) [22] solved this problem to some extent by using skip connections.

Inspired by the ResNet, Dense Convolutional Network (DenseNet) was proposed by Huang et al. [23]. By constructing dense blocks which adopt dense connections, DenseNet can deepen to more than 200 layers. Each layer in a dense block can directly access the gradient value from the loss function and the original input signal. By changing the growth rate, DenseNet can reduce the amount of parameters, but increase the computational cost [24].

### 2.2. SDFE Module and SSF-Nets

SAR images contain many different features from optical images. The traditional feature extraction methods need to consider the geometric features, statistical gray scale features, electromagnetic scattering features, transform domain features, local invariant features [25, 26], and so on. CNNs can adaptively learn the features of SAR images for recognition, which reduces the complexity of the recognition algorithm.

**Figure 2:**
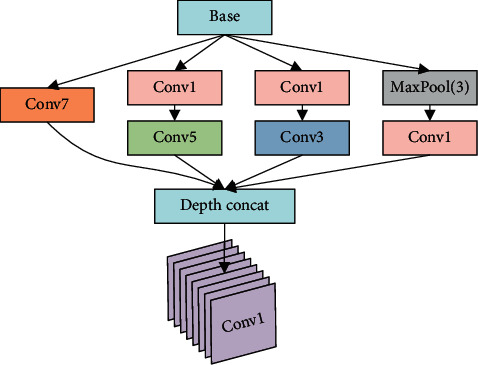
Structure of SDFE.

Although many studies have proved that, in the field of optical image recognition, deeper networks have better performance [22, 23]. However, the amount of SAR image data is relatively less. An overly complex network cannot significantly improve the recognition performance, and it may also carry the risk of overfitting. Therefore, the depth of the network proposed for SAR image recognition is not as deep as those of the ResNet and the DenseNet, so as to avoid the gradient disappearance problem that may appear in the late stage of training. The convolutional layer and pooling layer alternately and linearly propagate in our network. So, it can avoid skip connections to simplify the network complexity as much as possible. Due to the sparse feature of SAR images, when all the features are extracted by using small convolution filters, it may not be able to fully represent all the characteristics information of the target. Therefore, a Sparse Data Feature Extraction (SDFE) module is proposed in this paper, which contains a parallel convolution layer and a point convolution layer. Convolution filters with different sizes are introduced into the parallel convolutional layer to improve the ability of the network to extract sparse features in SAR images. The SDFE structure is shown in Figure 2, where “Conv7,” “Conv5,” “Conv3,” and “Conv1” represent convolutional layers with the filters size of 7 × 7, 5 × 5, 3 × 3, and 1 × 1, respectively. “MaxPool (3)” is the 3 × 3 max pooling layer with stride of 1.

The parallel convolutional layer of SDFE module utilizes 4 different filters with size of 7 × 7, 5 × 5, 3 × 3, and 1 × 1. The largest “7 × 7” convolutional filter in SDFE is crucial to improve the network's ability to extract feature from sparse data. The parallel convolutional layer in SDFE widens the network structure and further increases the depth of the network. The parallel convolutional layer is different from the Inception [27] module. In the Inception module, the largest convolutional filter size is 5 × 5, and following a point convolution layer, so its ability of sparse features extraction is limited. The SDFE parallel convolutional layer involves 7 × 7 convolution filters, and its input does not need to go through the point convolution layer to compress depth, which can directly extract features from the output of the upper network layer. The output of the parallel convolution module is followed by a point convolution layer after “depth concat”. The output depth of the point convolution layer is consistent with the input depth to increase the nonlinearity of the network and ensure that the SDFE module does not lose the feature information generated by the parallel convolution layer.

The large-scale convolution kernel can effectively extract the target features if the input data is sparse. The sparsity of the convolutional layer would bring many benefits, such as better robustness and higher feature extraction efficiency. However, if the input data is excessive sparse, feature extraction will become more difficult. Therefore, after repeated experiments, instead of the larger convolution kernel, the 7 × 7, 5 × 5, 3 × 3, and 1 × 1 filters are used in the parallel convolutional layer to alleviate this problem.

Based on the SDFE module, we propose 4 small sample sparse data feature extraction networks (SSF-Nets), as shown in Table 1. In Table 1, a SDFE structure is counted as two layers. The depth of the classifier in SSF-Net is set as 1. The “Conv” module in Figure 2 and Table 1 is a composite function containing “convolution,” “batch normalization,” and “activation function”.

AlexNet, VGGNets, and some other networks' classifiers are three Fully Connected layers (3-FC), which contain more than 80% of the parameters in the whole networks [16, 19] and need high memory requirements. RATR puts forward high requirements for real-time computing, and the recognition system should minimize the consumption of hardware. In order to reduce the amount of parameters and simplify the network, our network introduces one Fully Connected layers (1-FC) as classifier to concentrate the learning tasks into the convolutional layer and lighten the burden of the fully connected layer.

In addition, we introduce the Global Average Pooling (GAP) proposed by Lin et al. [28] to replace the FC layer as the classifier. This classifier does not require fully connected layers, which can greatly reduce the number of parameters and avoid overfitting problems in the SSF-Net under certain conditions. The SSF-Nets combined with the above three classifiers are represented by “SSF-NetX-GAP,” “SSF-NetX-1FC,” and “SSF-NetX-3FC,” where “X” indicates network's depth.

### 2.3. Network Complexity

If there are 4 types of targets, when using “3-FC” as the classifier, the size of output feature map generated by the last pooling (or convolution) layer of the network is H × W × D. The parameters in the classifier are calculated as follows:

(1) $$\begin{array}{c} 3-\text{FC}:\text{Parameters}=\text{H}\times \text{W}\times \text{D}\times 4096+4096+4096\times 4096+4096+4096\times 4+4\\ =16,801,796+4096\times \text{H}\times \text{W}\times \text{D}. \end{array}$$

When using the single layer fully connected layer “1-FC”, the parameters in the classifier is calculated as follows:

(2) $$\begin{array}{c} 1-\text{FC}:\text{Parameters}=\text{H}\times \text{W}\times \text{D}\times 4+4. \end{array}$$

When using “GAP” as the classifier, the global average pooling is used to replace the fully connected layer. Since the pooling layer has no parameters, it can further reduce the amount of parameters. The calculation formula is as follows:

(3) $$\begin{array}{c} \text{GAP}:\text{Parameters}=\text{D}\times 4+4. \end{array}$$

**Figure 3:**
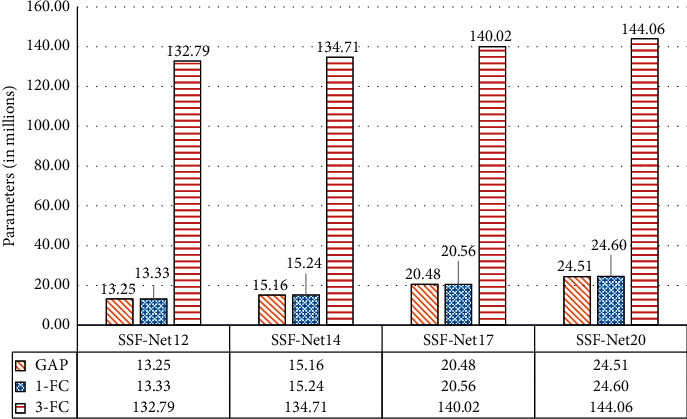
The parameters comparison of SSF-Net.

Through the above calculation, using the “1-FC” and “GAP” classifiers can save about 86%–92% of the parameters compared to that of the networks with “3-FC”, and the networks with “GAP” can further save about 100,000 parameters than the “1-FC” networks. The parameters of the SSF-Nets with different depths and different classifiers are shown in Figure 3.

It can be seen from Figure 3 that the type of the classifier has the greatest influence on the number of network parameters, followed by the network depth. As the network depth increases gradually, the amount of network parameters only increases slowly. If the RATR system hardware conditions are poor and the memory is insufficient, using “3-FC” as the network classifier would be a bad choice.

**Figure 4:**
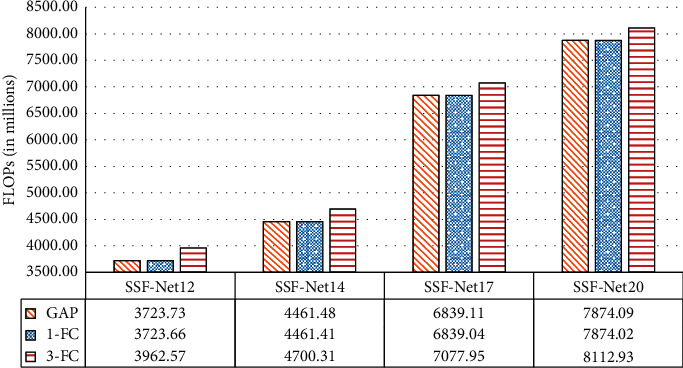
Comparison of floating points of operations (FLOPs).

Figure 4 shows the comparison of floating points of operations (FLOPs) of SSF-Net12, SSF-Net14, SSF-Net17, and SSF-Net20. According to Figure 4, the computation cost is most affected by the network depth. SSF-Net17 and SSF-Net20 are very computation-intensive. Compared to that of SSF-Net12, the FLOPs of SSF-Net14 has an increase of 19.82%. The FLOPs of SSF-Net17 has an increase of 53.31% compared to that of SSF-Net14, and the FLOPs of SSF-Net20 has an increase of 15.13% compared to that of SSF-Net17. So, if there is no significant difference in recognition accuracy, SSF-Net14 has the highest cost performance.

In addition, when the network depth is the same, the “3-FC” classifier has the highest computational cost, which is a fixed increase of 238.9 × 10^6^ compared to the other two classifiers. The calculation cost of the “1-FC” is the lowest, but it is not much different from “GAP”.

## 3. Experimental Results

### 3.1. Dataset

**Figure 5:**
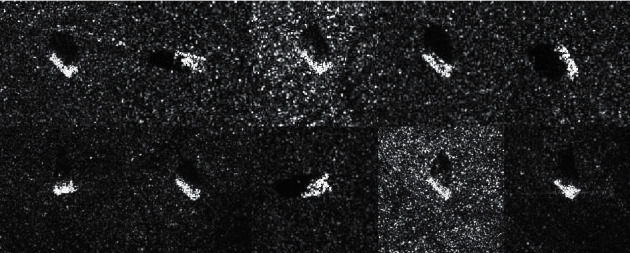
SAR images of MSTAR SAR-SOC dataset.

The Moving and Stationary target acquisition and recognition (MSTAR) dataset are used for the experiments. There are many research studies on radar automatic target recognition based on the MATAR SAR data set, such as [2–7, 10–12, 29]. The experimental results in this paper are compared with the above methods. The MSTAR dataset are classified into two datasets: Standard Operating Condition (SOC) dataset and Extended Operating Condition (EOC) dataset. In EOC-1 dataset, there are 4 kinds of ground targets, in which the targets with side view angle of 17° are used for training and the targets with side view angle of 30° are used for test. There are 10 kinds of targets in SOC dataset, each of which contains Omni-directional SAR image data at 15° and 17° pitch angles. In the experiments, observation data at 17° are used for training, and the observation data at 15° pitch angle are used for testing. The SAR images of MSTAR SAR-SOC dataset are shown in Figure 5.

SAR images are extremely sensitive to changes in pitch angle, so it is more difficult to identify the targets under EOC-1 conditions. The pitch angle difference between the SOC training set and test set is 2°, while the difference under the EOC-1 is increased to 13°. There is a big deviation of the same target in SAR images under the same posture, which increases the difficulty of recognition. The method in this paper has especially better recognition accuracy for SAR EOC-1 dataset and therefore has greater practical significance [7, 10, 30].

### 3.2. Preprocessing and Experiment Setup

In the experiments, each sample in the test set or the training set is resized to a fixed resolution of 128 × 128, and then the center cut and random horizontal rotation are performed. After this preprocessing, the number of SAR images has been expanded by 3 times, which compensates for the shortage of SAR images and alleviates the overfitting problem of the network to some extent.

In order to verify the validity of our approach, the experiments are completed on the same platform and environment, as shown in Table 2. The “batchsize” should be set to an appropriate value. Our original intention is to set the “batchsize” as large as possible within a suitable range to make the gradient calculation of the network more accurate. However, too large “batchsize” will make the model converge to the local optimum easily. Secondly, the “batchsize” is limited to the graphics card memory. After repeated experiments, we set the “batchsize” of the training set to 16 and that of the test set to 32.

Considering that the radar data is sparse, activation function Rectified Linear Unit (ReLU) [24] will undoubtedly increase this sparseness and reduce the useful information of the target, which is unfavorable for recognition. So, we use another activation function, Hyperbolic Tangent function (Tanh), as the activation function. The resulting impact will be further analyzed in the experiments.

The learning rate attenuation method is also introduced in the training processing. As the number of iterations increases, the learning rate gradually decreases. This can ensure that the model does not fluctuate greatly in the later period of training and closer to the optimal solution. After repeated experiments, the parameters are finally adjusted as follows: the initial learning rate is set as 0.01, and 200 epochs are used for training. The learning rate decreases by 2 times since the first 50 epochs and then decreased by 2 times every 20 epochs. The average recognition accuracy of the last 100 epochs is calculated as the final results.

### 3.3. Experimental Results

To focus on the impact of SSF-Net depth on recognition performance, we conducted experiments on the two MSTAR SAR datasets with the 4 depth SSF-Nets in Table 1 and the results are shown in Table 3.

According to Table 3, the recognition performance of SSF-Net12 is lower than that of the other 3 deeper networks. Because its structure is too simple to fully learn SAR image features for recognition, on SAR-EOC-1, SSF-Net14-3FC achieves the highest accuracy of 99.50%. The accuracies of SSF-Net14 with three different classifiers are 99.50%, 99.24%, and 99.05%, respectively, which are better than those of SSF-Net17 and SSF-Net20. On the SAR-SOC dataset, although SSF-Net17-GAP achieves the highest accuracy of 99.55%, most of the networks (except SSF-Net12) also achieve the accuracies higher than 99.3%. Because the difference of pitch angle between training set and test set of SAR-EOC-1 dataset is far greater than that of SAR-SOC dataset, the identification difficulty is greater, which requires the network to have strong generalization ability. Therefore, simply increasing the network depth does not significantly improve the networks' recognition performance, which also verifies that the excessively deep convolutional neural network is not conducive to SAR image recognition.

Based on the experimental results of SAR-EOC-1 in Table 3, we believe that SSF-Net14 has the best overall performance. SSF-Net14-1FC achieves 99.37% accuracy rates on SOC, only 0.18% lower than the highest accuracy achieved by SSF-Net14-3FC. On EOC-1, SSF-Net14-1FC also achieves 99.24% accuracy rates, only 0.26% lower than the highest accuracy achieved by SSF-Net17-GAP. “3-Fc” classifier has a large number of parameters and calculation, while “1-FC” classifier has a small number of parameters and calculation. Although “1-FC” has slightly more parameters than “GAP”, the computational cost is less. Next, we will compare the results of the SSF-Net14-1FC with GoogLeNet [27], ResNet-18 [18], and DenseNet-121 [19]. The results are shown in Table 4.

GoogLeNet achieves high recognition accuracies on SAR-SOC, but its recognition accuracies on SAR-EOC-1 are poor, which only 90.62% and 90.19%. This shows that its generalization ability is not so ideal. ResNet-18 and DenseNet-121 further deepen the network and apply skip connections to alleviate the gradient disappearance problem. However, the accuracy rates on SAR image recognition are still lower than that of our proposed network. Shallow networks have good capabilities of feature extraction and learning, so the networks with complex structures such as DenseNet-121 and ResNet-18 may bring overfitting problems to a certain extent. Based on horizontal comparison of the recognition accuracies of the activation functions, Tanh and ReLU in Tables 3 and 4, we can see the performance of Tanh on SAR-EOC-1 is generally stronger, indicating that Tanh has better effect on sparse data processing.

We further compare SSF-Net14-1FC with the methods proposed by Wang [10], Pei [11], and Chen [12], et al., and the results are shown in Table 5.

Although some methods such as A-CNN can achieve accuracy of 99.41% on the SAR-SOC, it is difficult to achieve satisfactory results on SAR-EOC-1 data which have greater difference in pitch angles. The 2DPCA-SCN method achieves 98.49% accuracy on SAR-EOC-1, but only 95.80% on SAR-SOC. Other methods on the SAR-EOC-1 also achieve lower recognition accuracies than SSF-Net. It can be found from Table 3 that SSF-Net achieves very high accuracy on both SAR-SOC and SAR-EOC-1 dataset. Especially on SAR-EOC-1 dataset, SSF-Net can achieve higher accuracy and more stable performance, which shows that our network has stronger generalization ability and better robustness.

SSF-Net is also compared with nondeep learning approaches (such as KNN, SVM, and SRC [7, 29]), and the results are shown in Table 6. Among them, “I-MTRL” is a new classification approach of clustering multitask learning theory. SRC [29] is a recognition approach based on Sparse Representation-based Classifier proposed in 2016.

Table 6 shows that some traditional approaches are not so effective, such as KNN and SVM methods. Although many complex classifiers have been designed, they cannot fully utilize the potential correlation between multiple radar categories. On the contrary, large-scale and complete SAR datasets are difficult to collect, so the samples obtained are usually limited or unbalanced. Traditional approaches are not able to share all the information, making it difficult to get good training results. Dong et al. [31] proposed a joint sparse representation model to take advantage of the correlation between multiple tasks of SAR ATR, and comparative experiments have demonstrated the superiority of multitask learning.

The classification algorithm approaches under the multitask framework has higher recognition accuracies, such as CMTL, MTRL, and I-MTRL. The multitask relational learning (MTRL) method proposed in [6] can autonomously learn the correlation between positive and negative tasks, and it can be easily extended to the nonlinear field. The MTRL is further improved by adding a projection regularization term to the objective function [7], which can independently learn multitask relationships, cluster information of different tasks, and can also be easily extended to nonlinear field. However, the Trace-norm Regularized multitask learning (TRACE), which is also under the multitask framework, has the lowest recognition accuracy. Because the TRACE method learns the linear prediction function and cannot accurately describe the nonlinear structure of SAR image, it also proves the importance of extending the multitask learning method to the nonlinear field.

The SSF-Net proposed in this paper can adaptively learn the nonlinear structure of SAR images and reduce the difficulty of redesigning the classifier when the SAR image conditions change. In contrast, the artificially designed feature extraction approach is complex, and sometimes it can only be effective for certain fixed problems. Its generalization ability is not so ideal. Therefore, our networks enhance the feature extraction capability of sparse data.

### 3.4. Experiments Analysis

SSF-Net17-GAP and SSF-Net14-3FC achieved the highest accuracy rates, 99.55% and 99.50%, on SAR-SOC and SAR-EOC-1 dataset, respectively. After a comprehensive selection, we compare the SSF-Net14-1FC with a variety of methods. It has achieved recognition accuracies, 99.37% and 99.24%, on SAR-SOC and SAR-EOC-1 dataset, which are higher than most of the accuracies achieved by other approaches.

By analyzing the different network structures and comparing the experimental results, the following conclusions are obtained:

1. The networks should not be too deep, and the structure should be as concise as possible. Due to the small amount of data in radar signal, some complex and deep networks, such as ResNets and DenseNets, may face the problem of overfitting.
2. Due to the sparsity of SAR images, large convolutional filters can be considered for feature extraction in the network. Different from the traditional sparse signal processing method [32], the SDFE module is designed to improve the network's ability to extract features from sparse data. However, the convolution filters in the first layer should not be too large. In this paper, we adopt 3 × 3 filters in the first convolution layer. Different from traditional optical images, SAR images do not have obvious edge features and texture information, so in the first layer, large-scale convolution filters cannot be used at quickly capture SAR image target edges and other features. On the contrary, the use of large-scale convolution filters at the first layer may cause excessive loss of detail information, which is not conducive to identification.
3. The network for SAR image targets recognition should increase the ability to learn nonlinear structures. Drawing on the view that the multitask learning method should be extended to the field of nonlinearity, the SDFE module increases the nonlinearity of the network with a point convolution layer that has no compression depth.

On SAR-EOC-1, Tanh has generally better performance. The main reason is that the SAR images have sparsity and the activation function ReLU may overenhance this nature. Excessively sparse data will weaken the ability of the convolutional layer to extract target features. And Tanh has a slightly better nonlinearity, so its performance is better when the original data features are significantly different. Overall, Tanh has better activation for radar signals.

## 4. Conclusions

In this paper, a feature extraction SDFE module and SSF-Net for sparse data is designed, which has good performance for radar targets recognition.

One of the advantages of SSF-Net is that it can achieve high accuracy on both SAR-SOC and SAR-EOC-1. On SAR-SOC, the accuracy rate of SSF-Net14-1FC has only 0.18% lower than the highest accuracy rate achieved by the SSF-Net17-GAP. However, it saves 25.84% parameters and 34.77% FLOPs than SSF-Net17-GAP. On SAR-EOC-1, the accuracy rate of SSF-Net14-1FC is only 0.26% lower than the highest accuracy rate, but it saves more than 88.6% of the parameters. SSF-Net14-1FC saves at least 36.97% FLOPs than SSF-Net17-3FC and SSF-Net20-GAP. Therefore, SSF-Net can achieve better recognition performance for SAR images with a shallow network, improves the computational efficiency, and saves parameter space.

The SDFE module, as the most important part in SSF-Net, has three advantages. Firstly, the SDFE module can effectively extract the target features when the input data is sparse. Secondly, the SDFE module improves the nonlinearity of SSF-Net, which can strengthen the SSF-Net's ability to fit the nonlinear structure of SAR images. Lastly, the SDFE module increases the robustness and computational efficiency of SSF-Net, so the SSF-Nets can achieve high accuracies on SAR-EOC-1 with fewer layers.

When deepening the network, the recognition algorithm may be invalid. It is because the down-sampling layers in the deep neural network are too many for SAR images. To solve this problem, one feasible method is to reduce the down-sampling layers of the deep neural network, but it will weaken the robustness of the network and increase the computational cost. Another solution is to design shallow convolutional neural networks, such as our SSF-Nets proposed in this paper.

According to the imaging characteristics of SAR images, another feasible method to improve the target recognition rate is target classification and recognition based on image superresolution reconstruction [33], which is also a key research direction at present.

## Figures and Tables

**Table 1:** SSF-Net configuration.

SSF-Net12	SSF-Net14	SSF-Net17	SSF-Net20
conv3-64	conv3-64	conv3-64	conv3-64
	conv3-64	conv3-64	conv3-64
2 × 2 MaxPool, stride:2			
conv3-128	conv3-128	conv3-128	conv3-128
	conv3-128	conv3-128	conv3-128
2 × 2 MaxPool, stride:2			
**SDFE-256**	conv3-256	conv3-256	conv3-256
Conv3-256	**SDFE-256**	**SDFE-256**	**SDFE-256**
		conv3-256	conv3-256
			conv3-256
2 × 2 MaxPool, stride:2			
**SDFE-512**	conv3-512	conv3-512	**SDFE-512**
**SDFE-512**	conv3-512	**SDFE-512**	conv3-512
		**SDFE-512**	conv3-512
			**SDFE-512**
2 × 2 MaxPool, stride:2			
conv3-512	**SDFE-512**	conv3-512	conv3-512
conv3-512	**SDFE-512**	conv3-512	conv3-512
		conv3-512	conv3-512
			conv3-512
2 × 2 MaxPool, stride:2			
Classifier, soft-max			

**Table 2:** Experimental platform configuration.

Attribute	Configuration information
OS	Ubuntu 14.04.5 LTS
CPU	Intel(R) Xeon(R) CPU E5-2670 v3 @ 2.30 GHz
GPU	GeForce GTX TITAN X
CUDNN	CUDNN 6.0.21
CUDA	CUDA 8.0.61
Framework	PyTorch

**Table 3:** Recognition accuracy rates of different depth SSF-Nets (%).

Method	SAR-SOC		SAR-EOC-1	
	Tanh	ReLU	Tanh	ReLU
SSF-Net12-3FC	98.49	99.19	95.32	97.55
SSF-Net12-1FC	97.47	99.09	97.02	96.58
SSF-Net12-GAP	99.33	98.99	97.17	97.02
SSF-Net14-3FC	99.27	99.34	**99.50**	98.59
SSF-Net14-1FC	**99.37**	99.20	**99.24**	97.96
SSF-Net14-GAP	99.18	99.43	99.05	97.55
SSF-Net17-3FC	99.39	99.37	99.36	98.92
SSF-Net17-1FC	99.31	99.35	98.81	98.02
SSF-Net17-GAP	**99.55**	**99.45**	98.78	95.67
SSF-Net20-3FC	99.43	99.35	98.47	**99.16**
SSF-Net20-1FC	99.54	99.34	98.69	98.63
SSF-Net20-GAP	99.42	99.30	99.33	98.11

**Table 4:** Recognition accuracy rates of other CNNs (%).

Method	SAR-SOC		SAR-EOC-1	
	Tanh	ReLU	Tanh	ReLU
GoogLeNet	98.87	98.65	90.62	90.19
ResNet-18	97.20	97.90	78.45	82.25
DenseNet-121(*k* = 32)	98.66	98.93	96.41	98.66
SSF-Net14-1FC	**99.37**	99.20	**99.24**	97.96

**Table 5:** Recognition accuracy rates of other CNNs (%).

Method	SAR-SOC	SAR-EOC-1
2DPCA-SCN [10]	95.80	98.49
2-Views DCNNs [11]	97.81	93.29
3-Views DCNNs [11]	98.17	94.34
4-Views DCNNs [11]	98.52	94.61
A-CNN [12]	**99.41**	97.13
SSF-Net14-1FC	**99.37**	**99.24**

**Table 6:** Recognition accuracies rate of traditional approaches (%).

Method	SAR-SOC	SAR-EOC-1
KNN [2]	92.71	91.42
SVM [2]	90.17	86.73
SRC [29]	89.76	—
TRACE [3]	75.04	67.42
RMTL [4]	92.09	92.03
CMTL [5]	93.91	94.72
MTRL [6]	95.84	95.46
I-MTRL [7]	97.34	98.24
SSF-Net14-1FC	**99.37**	**99.24**

## Data Availability

All datasets in this article are public datasets and can be found on public websites.
